# Laparoscopic adhesiolysis: not for all patients, not for all surgeons, not in all centres

**DOI:** 10.1007/s13304-018-0534-4

**Published:** 2018-05-16

**Authors:** Salomone Di Saverio, Arianna Birindelli, Richard Ten Broek, Justin R. Davies, Matteo Mandrioli, Ville Sallinen

**Affiliations:** 1 Emergency and Trauma Surgery Unit, Maggiore Hospital Regional Emergency Surgery and Trauma Center-Bologna Local Health District, Bologna, Italy; 2 Addenbrookes Hospital, CUH NHS Trust-University of Cambridge, Cambridge, UK; 3 Queen Elizabeth Hospital, University of Cambridge, Cambridge, UK; 4 Department of Surgery, Radboud University Nijmegen Medical Center, Nijmegen, The Netherlands; 5 Department of Abdominal Surgery, Helsinki University Central Hospital, Haartmaninkatu 4, 00029 Helsinki, Finland

**Keywords:** Abdominal adhesions, Adhesive small bowel obstruction, Bowel injury, Bowel resection, Laparoscopy, Small bowel obstruction, Laparoscopic adhesiolysis

## Abstract

ASBO is a common cause of emergency surgery and the use of laparoscopy for the treatment of these patients is still under debate and conflicting results have been published, in particular regarding the high risk of iatrogenic bowel injury. In fact, although over the last few years there has been an increasing enthusiasm in the surgical community about the advantages and potential better outcomes of laparoscopic management of adhesive small bowel obstruction (ASBO), recently published studies have introduced a significant word of caution. From 2011 in our centre, we have started to systematically approach ASBO in carefully selected patients with a step-by-step standardized laparoscopic procedure, developed and performed by a single operator experienced in emergency laparoscopy, collecting data in a prospective database. Inclusion criteria were: stable patients (without diffuse peritonitis and/or septic shock with suspicion of bowel perforation), CT scan findings consistent with a clear transition point and therefore suspected to have a single obstructing adhesive band. Patients with diffuse SB distension in the absence of a well-defined transition point and suspected to have diffuse matted adhesions (based on their surgical history and radiological findings) should be initially managed conservatively, including gastrografin challenge. Up to date, 83 patients were enrolled in the study. The rate of iatrogenic full-thickness bowel injury was 4/83 (4.8%); two of these cases were managed with simple repair and the other two required bowel resection and anastomosis. Conversion to open was performed in 3/4 of these cases, whereas in one a repair of the full-thickness injury was completed laparoscopically. All the iatrogenic injuries were detected intraoperatively and none of the reoperations that occurred in this series were due to missed bowel injuries. At 30 days follow-up, none reported incisional hernias or SSI or death. With the described accurate selection of patients, the use of such standardized step-by-step technique and in the presence of dedicated operating surgeons with advanced emergency surgery laparoscopic expertise, such procedure can be safe and feasible with multiple advantages in terms of morbidity and LOS. A careful preoperative selection of those patients who might be best candidates for laparoscopic adhesiolysis is needed. The level of laparoscopic expertise can also be highly variable, and not having advanced surgical expertise in the specific subspecialty of emergency laparoscopy, ultimately resulting in performing standardized procedures with proper careful and safe step-by-step technique, is highly recommended.

Dear Editor,

We read with great interest the study by Behman et al. on laparoscopic adhesiolysis [1]. Despite increasing enthusiasm for laparoscopic management of adhesive small bowel obstruction (ASBO), this study introduces significant caution and raises serious concern for the proposed laparoscopic approach to ASBO due to a reported higher risk of bowel injury from a population-based analysis of more than 8500 patients. We share the same concerns in recommending to all general and acute care surgeons in every centre, a routine laparoscopic approach for all patients with ASBO. In fact, in this manuscript a routine laparoscopic approach seems to have been used regardless of patients’ characteristics and surgeons’ expertise.

Firstly, careful preoperative selection of those patients who might be best suited for laparoscopic adhesiolysis is needed; the level of laparoscopic expertise may be highly variable, and having advanced surgical expertise in the specific subspecialty of emergency laparoscopy [2], leading to standardized procedures with a safe step-by-step technique, is highly recommended before undertaking such procedures.

We have started to systematically approach ASBO in carefully selected patients from 2011 with a step-by-step standardized laparoscopic procedure, developed and performed by a single operator experienced in emergency laparoscopy, collecting data in a prospective database [3]. Up to May 2017, we have treated a prospective consecutive series of 83 cases, all operated by the same single operator with a standardized step-by-step technique developed by the same surgeon. We are also involved as co-investigators of the Finnish trial on laparoscopic vs open adhesiolysis for ASBO [4]. From our previous experience in the field, we have developed and adopted a well-defined protocol for laparoscopic management of ASBO. The selection of patients must be accurate and only stable patients (without diffuse peritonitis and/or septic shock with suspicion of bowel perforation) having CT scan findings consistent with a clear transition point and therefore suspected to have a single completely obstructing adhesive band should be considered for the laparoscopic approach [5]. Patients with diffuse SB distention in the absence of a well-defined transition point and suspected to have diffuse matted adhesions (based on their surgical history and radiological findings) should be initially managed conservatively, including a Gastrografin challenge [6].

In detail, the inclusion and exclusion criteria in our experience were:

Inclusion criteria:

- adult patients;
- informed consent;
- initial CT diagnosis of complete ASBO with an identifiable transition point and an anticipated single obstructing band with completely collapsed distal small bowel loops;
- and/or with a radiological/clinical evidence of failure of a NOM trial with hyperosmolar WSCM via NGT.

Exclusion criteria:

- hemodynamic instability and preoperative shock;
- diffuse peritonitis and/or evidence of severe intra-abdominal sepsis;
- high suspicion of gangrenous/perforated bowel;
- high probability of diffuse and dense matted adhesions (e.g. multiple previous laparotomies ≥ 3 with intraoperative findings of diffuse dense and matted adhesions);
- preoperative diagnosis of any other cause of complete mechanical SBO than adhesions (i.e. carcinomatosis, hernias, cancer, intussusception, biliary ileus, etc.).

The following were NOT exclusion criteria from diagnostic laparoscopy in our experience, although these are risk factors for conversion after an initial diagnostic laparoscopy:

- previous midline laparotomy was not considered an absolute contraindication;
- suspicion of bowel strangulation and/or volvulus and/or bowel ischaemia without gangrene or perforation;
- localized clinical peritonitis;
- CT findings of free abdominal fluid;
- CT or AXR findings of small bowel distension with a size > 4 cm;
- CT additional findings such as SB faeces sign, SB thickening, and mesenteric oedema/vascular engorgement.

Careful interpretation of the CT scan findings may be useful in identifying good candidates for a laparoscopic approach. We also recommend that only fully trained and experienced laparoscopic surgeons should attempt this technique. The correct surgical technique is of paramount importance to avoid bowel injuries. From our experience, once a laparoscopic approach is decided, we recommend not to use Veress needles or blind insertion of the first port in close proximity to the previous scars. We believe that a safe entry to the abdomen can be best obtained by either inserting the first Hasson trocar in the left flank with open access or by using a blunt dilating tip, optical trocar entering the abdominal wall at the level of the Palmer point, under direct vision (step 1—Fig. 1) [7]. Once the pneumoperitoneum has been gradually established, the surgeon must assess if there is adequate room for good vision and further safe trocar placement. If not, we would recommend timely conversion. Inadequate vision mandates withdrawing from any attempt to manipulate the bowel using laparoscopic instruments.

**Fig. 1:**
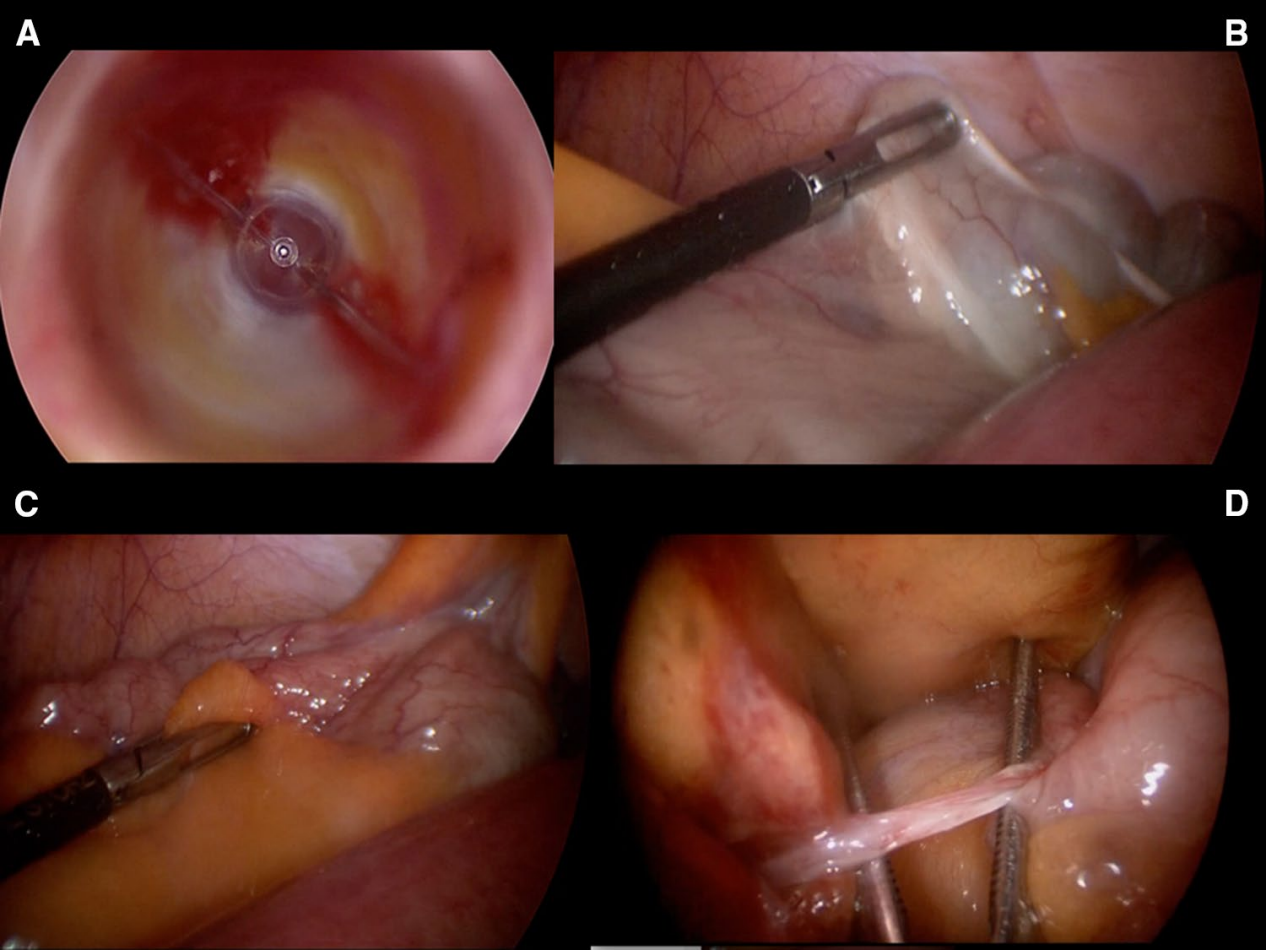
How to do it step-by-step a safe laparoscopic lysis of adhesions: **a** step 1: entrance with blunt dilating tip optical trocar at the level of the Palmer’s point, under direct vision; **b** step 2: identification of the caecum and ileo-caecal valve; **c** step 3: running the bowel from the collapsed distal ileal loop in a distal-to-proximal fashion; **d** step 4: identification of the transition point and the single obstructing band

Further tips and tricks derived from our experience are not to immediately search for the transition point and for the strangulating band by manipulating the distended and fragile bowel loops, but rather to start with identification of the caecum, then starting to explore from the collapsed distal ileal loop in a distal-to-proximal fashion (step 2—Fig. 1). Only collapsed loops should be manipulated. Grasping the mesentery rather than the bowel wall helps to run the bowel without traumatizing the bowel (step 3—Fig. 1). Usually, the distal collapsed loops can be run relatively easily and explored until reaching what we used to name the “sentinel loop”, a collapsed loop which is fixed and stuck, giving the feeling to the operator that running the bowel cannot be continued further proximally. Usually, this is the location for the transition point; by carefully grasping only the mesentery of the dilated loop adjacent to this point, the level of transition between the proximal distended bowel and the distal collapsed loop is identified and often the single obstructing band can be seen (step 4—Fig. 1). The advice at this point is to gently underpass the band with the aid of blunt manoeuvres, for example using the suction device first and/or spreading the two branches of an atraumatic grasper. These manoeuvres allow one to visualize and isolate the band and at the same time to obtain a little space from the adjacent bowel loops. Once a window is obtained, the band can be carefully and easily cut using cold scissors over the guidance of the two open branches of the atraumatic grasper which are spread and used in a fashion of a right angle instrument (step 5—Fig. 2). A further strong recommendation is to avoid any use of energy-based dissection, either monopolar or bipolar, during the procedure. Thermal injuries may evolve to delayed perforation only several days afterwards.

**Fig. 2:**
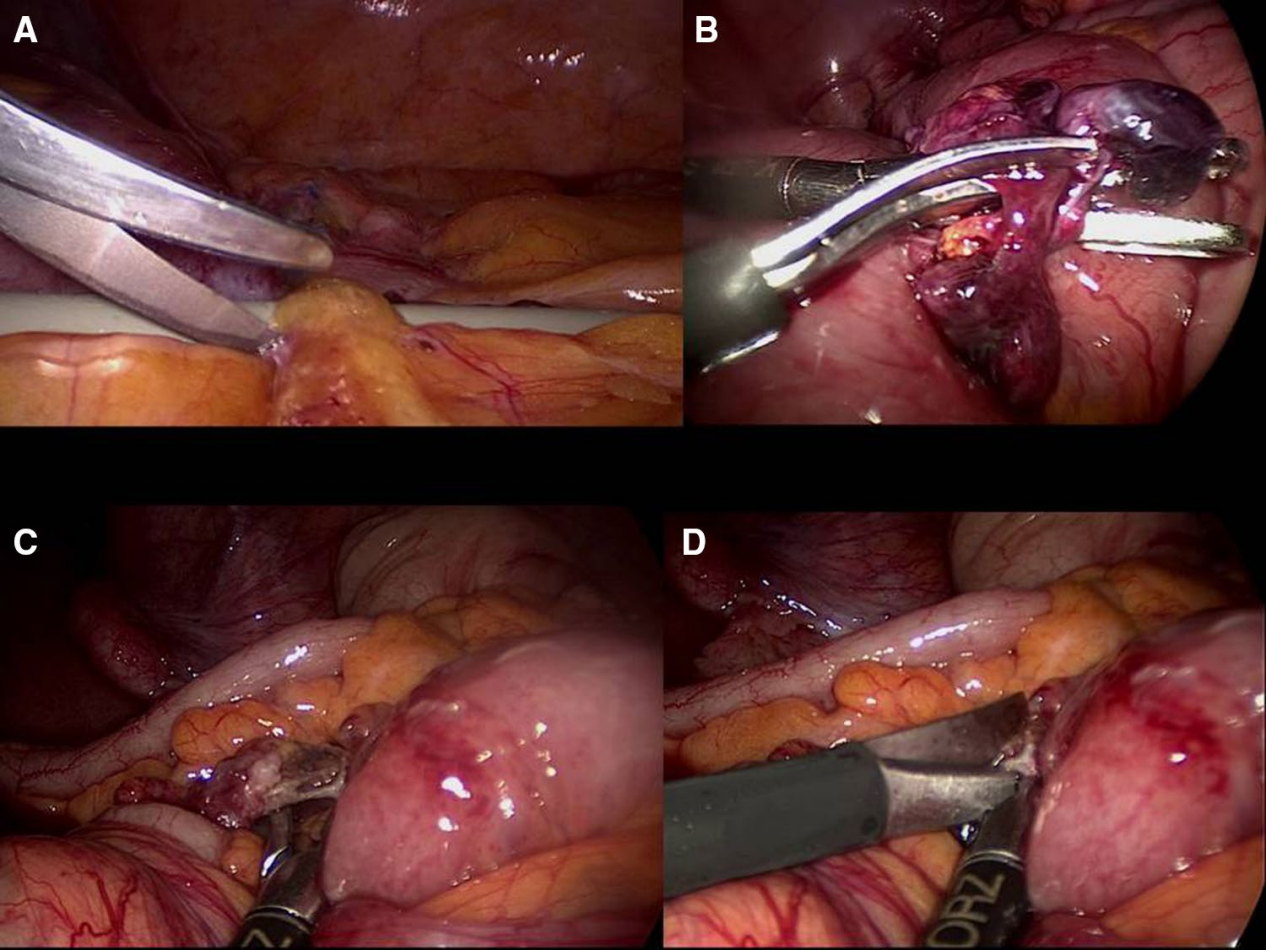
Step 5: gently underpassing the band with the aid of blunt manoeuvres using the suction device first and/or spreading the two branches of an atraumatic grasper (**a**, **c**). These manoeuvres allow visualization and isolation of the band whilst obtaining a little space from the adjacent bowel loops. Once a window is obtained, the band can be carefully and easily cut using cold scissors over the guidance of the two open limbs of the atraumatic grasper which are spread and used in the fashion of a right angle instrument (**b**, **d**)

Any bleeding during lysis of adhesions is generally minor and often self-limiting. Gauze compression may help and any persistent bleeding can be dealt with at the end of the operation, after releasing the obstruction and before finishing the procedure. Finally, adhesiolysis should be limited to only the obstructing band or to the adhesions that need to be divided to reach the transition point. Further extensive adhesiolysis is unnecessary, potentially harmful and should therefore be avoided. Careful observation for any serosal tears over the loops which were manipulated may allow intraoperative laparoscopic simple repair of these, thus preventing postoperative fistula or leak. Full-thickness injury usually mandates conversion. In our previously mentioned prospective single-operator series (unpublished data), the current rate of iatrogenic full-thickness bowel injury is 4/83 (4.8%); two of these cases were managed with simple repair and the other two required bowel resection and anastomosis. Conversion to open was performed in 3/4 of these cases, whereas in one a repair of the full-thickness injury was completed laparoscopically. All the iatrogenic injuries were detected intraoperatively and none of the reoperations that occurred in this series were due to missed bowel injuries.

With the described accurate selection of patients, the use of such standardized step-by-step technique [8] and in the presence of dedicated operating surgeons with advanced emergency surgery laparoscopic expertise and experience in the field of laparoscopic adhesiolysis, such procedure can be safe and feasible with multiple advantages in terms of morbidity and LOS [9–11].

In the study by Behman, patient selection criteria are not specified since the study is a population-based cohort study where data have been collected from health administrative records. However, some degree of patient selection emerges from the study, since patients undergoing laparoscopic procedures were younger, had fewer comorbidities and were cared for at larger hospitals.

We therefore agree with the authors’ conclusions that surgeons should approach laparoscopic adhesiolysis with a high level of awareness and use strategies to mitigate the risks. We suggest that laparoscopic adhesiolysis is not for all patients and may not be for all surgeons. Ideally, laparoscopic adhesiolysis should be only performed in high volume centres with specific expertise in emergency laparoscopy, especially in tertiary referral centres with availability of operating room and laparoscopic equipment at all times, and with highly trained surgical and OR staff.

As investigators of the current Finnish–Italian LASSO trial4, we eagerly await the results and will hopefully clarify further details about safety and outcomes of the laparoscopic treatment of ASBO.
